# Knowledge domain and trend of disease-modifying therapies for multiple sclerosis: A study based on CiteSpace

**DOI:** 10.1016/j.heliyon.2024.e26173

**Published:** 2024-02-17

**Authors:** Ting Zheng, Taotao Jiang, Zilong Huang, Manxia Wang

**Affiliations:** a Department of Neurology, The Second Hospital & Clinical Medical School, Lanzhou 730000, PR China; b The Second Clinical Medical School, Lanzhou University, Lanzhou 730000, PR China

**Keywords:** Multiple sclerosis, Disease-modifying therapy, CiteSpace

## Abstract

**Objective:**

To explore the current status and trends of disease-modifying therapies (DMTs) for multiple sclerosis through bibliometric and visual analyses of the related literature.

**Methods:**

Relevant literature from the Web of Science Core Collection from 2017 to 2022 was retrieved, and a bibliometric analysis was performed using CiteSpace 6.1. R2. Thesoftware was used to generate visual graphs of the author, institution, country, keyword co-occurrence, and literature co-citation network.

**Results:**

A total of 1719 manuscripts were retrieved, including 1397 original studies and 322 reviews. In the past five years, Patti F and the University of London were the authors and institutions generating the largest number of publications, respectively, and there was active collaboration between authors and institutions. The United States was the largest contributor to the relevant literature, and the high-frequency keywords in the field of multiple sclerosis disease-modifying therapies in the past five years mainly included multiple sclerosis, disease-modifying therapy, double-blind, disability, natalizumab, effectiveness, fingolimod, glatiramer acetate, and dimethyl fumarate.

**Conclusions:**

Current research hotspots and trends in DMTs in multiple sclerosis focus on the effectiveness of different DMTs drugs in treating patients with MS and how to optimise treatment strategies. In the context of the COVID-19 pandemic, the correlation between MS and COVID-19 infection and the method to manage and address the adverse effects of DMTs on multiple sclerosis patients is also future research trends.

## 1 Introduction

Multiple sclerosis (MS) is a chronic immune-mediated demyelinating disease of the central nervous system. It is the leading cause of disability in young adults worldwide and has tremendous socioeconomic impact. Nearly 2.8 million patients have multiple sclerosis worldwide, with an expected increase in cases in the future [1,2]. The pathological changes in MS mainly involve damage to the central nervous system by overreactive immune responses, leading to paroxysmal attacks and neurodegeneration, contributing to progressive deterioration. Disease modification therapies (DMTs) have been one of the most significant revolutionary inventions in treating MS over the last 20 years, particularly in the last 5 years. They reduce disease recurrence and progression by regulating immunity, slowing disease progression, and promoting functional reconstruction [3]. A growing number of studies have found that using DMTs may reduce the progression of disability and the relapse rate of multiple diseases and increase the life span of MS patients. As a promising treatment strategy for MS, efforts dedicated to developing DMTs have increased over the past 5 years. However, concerns remain regarding the side effects of DMTs, such as lymphopenia and increased infection. In addition, secondary autoimmune diseases, particularly progressive multifocal leucoencephalopathy (PML), draw public attention and limit the clinical application of DMTs [4]. This scientometric study aimed to summarise the treatment of DMTs in the field of MS, which will help analyze the research situation of DMTs in MS and provide a consultative opinion for future research on the treatment of MS.

This study applied a bibliometric strategy based on literature sources from the authoritative Web of Science Core Collection database. We collected all relevant research involving DMTs in MS in the past 5 years (2017–2022). Bibliometrics is a common analytical method for evaluating or quantifying research literature and information. CiteSpace, developed by Professor Chen Chaomei in 2004, is a web-based Java application primarily used to explore research progress and trends in the field of knowledge [5]. To our knowledge, no bibliometric research analyzes systematic data about DMTs in MS. This study uses CiteSpace to reveal the trends of publications and citations, highly cited authors, institutions, countries, journals, and hotspots in this field.

## 2 Materials and methods

### 2.1 Databases for literature

The data for the bibliometric analysis were obtained from the Web of Science Core Collection, including SCIEXPANDED, SSCI, A&HCI, CPCI–S, CPCI-SSH, BKCI–S, BKCI-SSH, ESCI, CCR-EXPANDED, and IC. Keywords used include 'multiple sclerosis' and 'disease-modifying therapy' or 'disease-modifying therapies' on DMTs treatment of MS published between January 1st, 2017 and July 1st, 2022. In total, 1719 articles were extracted, including 1397 original articles and 322 reviews.

### 2.2 Inclusion criteria

The inclusion criteria were peer-reviewed original research on MS's DMTs field, including clinical, basic research, and related reviews; articles published from January 1st, 2017 to July 1st, 2022; and articles retrieved from the Web of Science database.

### 2.3 Exclusion criteria

Studies excluded from the analysis included those collected by hand or telephone, articles not officially published, conference abstracts and proceedings, corrigendum documents, repeated publications, and unrelated articles.

### 2.4 Quantitative analysis methods

CiteSpace 6.1R2 was employed for visual analysis. The software's parameters utilized were as follows: the time zone spanned from January 1st, 2017 to July 1st, 2022, with a time slice of "1"; the term source encompassed "title," "abstract," "author," and "keywords"; the node types encompassed author, organization, keywords, and others; the TopN% value was set at 50, while the remaining parameters adhered to the default software settings. The outcome of the analysis generated authors, institutions, keywords, clustering modules, and visual maps pertinent to the emerging field of MS's DMTs research. In the visual maps, cooler colors denote earlier years, while warmer colors denote more recent ones.

## 3 Results

### 3.1 Analysis of publication years and journals

**Fig. 1:**
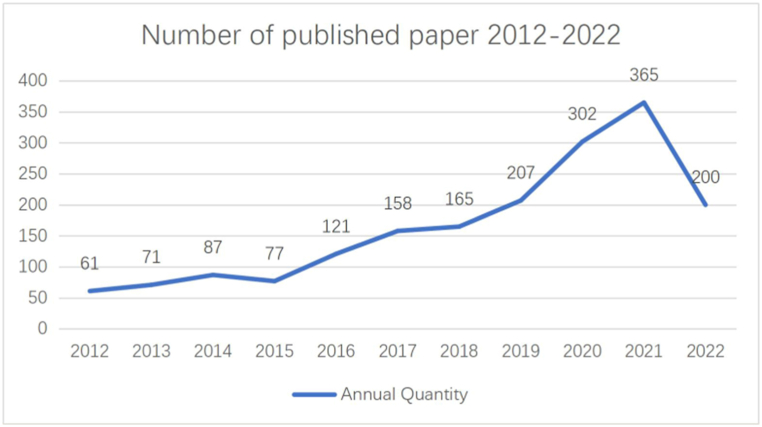
Time sequence of relevant papers on DMTs for MS published from 2017 to 2019 in Web of Science.

Since the approval of the first DMT drug interferon-β1b for the treatment of MS in 1993, more than 10 different types of DMTs drugs have been approved for the treatment of MS. However, few kinds of DMTs drugs are available for MS before 2010. In addition to interferon-β1b, a variety of drugs such as interferon-β1a,peginterferon beta-1a, glatiramer acetate, teriflunomide, dimethyl fumarate, fingolimod, siponimod, ozanimod, cladribine, natalizumab, alemtuzumab, ocrelizumab, and ofatumumab have been approved [6,7]. The number of articles published in DMTs research for MS in the last ten years shows that the number of studies has increased significantly in the last five years compared to the number of articles published from 2012 to 2016. The number of manuscripts increased from 121 in 2016 to 365 in 2021 (Fig. 1). During the first half of 2022, 200 articles were published. This rapid growth rate shows that increasing attention has been drawn to DMTs as a crucial therapeutic strategy for MS.

Literature on MS in the field of DMTs research has been published in various journals. Table 1 presents the top ten journals based on the number of publications. The number of articles published in the journal Multiple Sclerosis and Related Disorders was the highest in the past five years, reaching 219. This journal aims to promote an understanding of the aetiology, pathogenesis, epidemiology, genetics, treatment, and psychology related to MS. Since its inclusion in the Web of Science in 2012, 3283 papers have been published in the core collection of Web of Science, with 25,276 citations. The H-index was 52, and the influence factor was 4.808. The journal *Multiple Sclerosis* ranks second in terms of the number of published articles, with 96 articles. The average annual number of articles published was 223 since it was published in 1995 and indexed by the Web of Science in 1997. By now, 5410 papers have been published and cited 26,622 times, with an average citation of 4.92 times. The H-index was 79, and the influencing factor was 5.855.

**Table 1:** Top 10 journals by number of publications 2017–2022.

Journals	publication	IF
Multiple Sclerosis and Related Disorders	219	4.808
Multiple Sclerosis	96	5.855
Journal of Neurology	50	6.682
Frontiers in Neurology	46	4.086
Multiple Sclerosis Journal-Experimental Translational and Clinical	33	2.566
Therapeutic Advances in Neurological Disorders	28	6.43
Neurology	25	11.8
Neurology-Neuroimmunology & Neuroinflammation	23	11.36
CNS Drugs	21	6.497
BMC Neurology	20	2.903

### 3.2 Visual analysis of Co-country

**Fig. 2:**
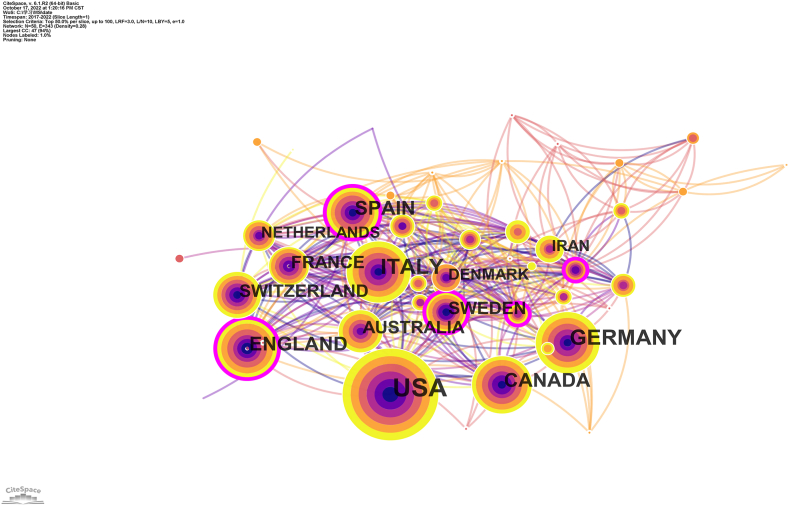
Co-country of Disease-modifying therapies for multiple sclerosis.

In the visual graph generated by the CiteSpace software, the node's centrality represents the node's connection function in the entire network, which measures the number of shortest paths through the node in the network. The more it appears on the shortest path in the entire network, the greater the centrality of a node and hence, the higher its influence and importance [5].The co-country results for the MS DMTs research field are shown in Fig. 2. The circles in the figure represent the number of papers published in each country. The shorter the distance between the two circles, the greater the cooperation between the two countries. The purple ring in Fig. 2 indicates that these countries have a greater intermediary centrality (not less than 0.1) and greater importance. From 2017 to 2022, 87 countries published papers related to MS's DMTs research field. The United States, Italy, Germany, England, and Canada are the top five countries in the field of MS DMTs research (Table 2), and the United States has a large intermediary centre. Thus, it can be deduced that there is a large gap in terms of research conducted among different countries and that the United States has contributed the most to MS DMTs research (see Table 3).

**Table 2:** Top 10 countries for disease-modifying therapies for multiple sclerosis.

Rank	Country	Publication	Proportion (%)
1	The United States	504	36.08
2	Italy	217	15.53
3	Germany	177	12.67
4	England	144	10.31
5	Canada	135	9.66
6	Spain	110	7.87
7	Switzerland	91	6.51
8	Australia	83	5.94
9	France	72	5.15
10	Sweden	62	4.43

**Table 3:** The top 10 articles with the most citations for Disease-modifying therapies for multiple sclerosis.

Title	First Author	Year	Citation	Journal
Ocrelizumab versus Interferon Beta-1a in Relapsing Multiple Sclerosis	Hauser SL	2017	170	NEJM
Diagnosis of multiple sclerosis: 2017 revisions of the McDonald criteria	Thompson AJ	2018	161	Lancet Neurology
Ocrelizumab versus Placebo in Primary Progressive Multiple Sclerosis	Montalban X	2017	130	NEJM
Practice guideline recommendations summary: Disease-modifying therapies for adults with multiple sclerosis: Report of the Guideline Development, Dissemination, and Implementation Subcommittee of the American Academy of Neurology	Rae-Grant A	2018	126	Neurology
Siponimod versus placebo in secondary progressive multiple sclerosis (EXPAND): a double-blind, randomised, phase 3 study	Kappos L	2018	77	Lancet
Defining the clinical course of multiple sclerosis: the 2013 revisions	Lublin FD	2014	77	Neurology
Multiple Sclerosis	Reich DS	2018	76	NEJM
ECTRIMS/EAN Guideline on the pharmacological treatment of people with multiple sclerosis	Montalban X	2018	71	Multiple Sclerosis
Safety and efficacy of fingolimod in patients with relapsing-remitting multiple sclerosis (FREEDOMS II): a double-blind, randomised, placebo-controlled, phase 3 trial	Calabresi PA	2014	67	Lancet Neurology
Oral teriflunomide for patients with relapsing multiple sclerosis (TOWER): a randomised, double-blind, placebo-controlled, phase 3 trial	Thompson AJ	2018	62	Lancet Neurology

NEJM:The New England Journal of Medicine.

### 3.3 Visual analysis of Co-institute

**Fig. 3:**
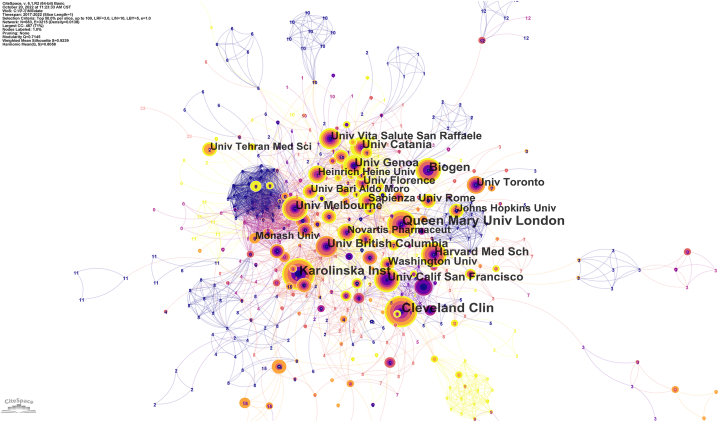
Co-institute of Disease-modifying therapies for multiple sclerosis.

When the research institutions of the two authors appeared in the same article, they were recognised as cooperating between the two research institutions. A co-occurrence frequency matrix was used to visualise whether the institutions cooperated. In the past five years, MS's DMTs research field has involved 2541 institutions, of which 184 have published more than 10 articles. Many institutions are involved in the DMTs studies of MS. The results are displayed in Fig. 3. The nodes in the map represent research institutions, and each node's size corresponds to the research institutions' co-occurrence frequency. The circle size represents the number of papers published by the research institution; the shorter the distance between the two circles, the greater the cooperation between the research institutions. As shown in Fig. 3, there is a close cooperative relationship among institutions, and the University of London, Cleveland Clinic, and the University of California are among the top three institutions in terms of publication duration.

### 3.4 Visual analysis of Co-authorship

**Fig. 4:**
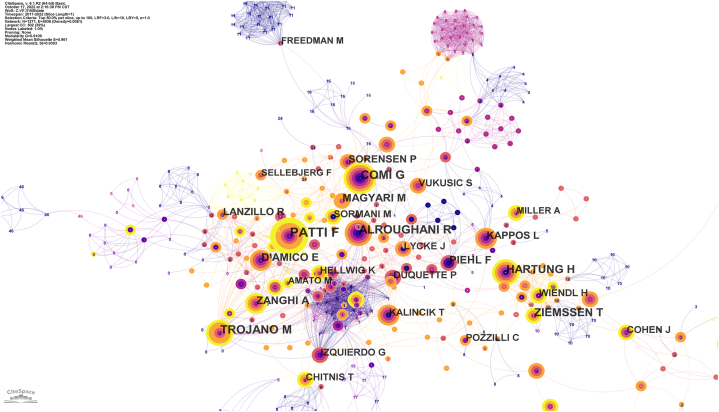
Co-authorship of Disease-modifying therapies for multiple sclerosis.

The analysis of the author's cooperative relationship network graph can provide other researchers with concrete scientific research information, including the number of articles published by each researcher, the research direction, and the relationship of mutual cooperation. Fig. 4 depicts the MS collaborative network of authors in the field of DMTs research. Each node represents an author, and the size of the circle in the graph represents the number of studies published by the author. The shorter the distance between the two circles, the closer the cooperation between the two authors. Authors in the graph with the same number of nodes are within the same cluster, indicating active communication and cooperation between them. As shown in Fig. 4, researchers tend to have long-term and stable communication and cooperation with others, represented as aggregations in the graph; each aggregation includes several core researchers. These researchers made significant contributions to the field of MS DMTs. The most authoritative author with the largest number of publications in the field of DMTs in MS was Patti F, with 35 publications in the last five years. Patti et al. published studies on the effectiveness and relapse rate of DMTs such as interferon-β1 [8,9], dimethyl fumarate [10], natalizumab [11], alemtuzumab [12], and teriflunomide [13] in patients with MS, and the risk of COVID-19 infection in MS patients [14]. For Patti F,428 papers were published, with 4439 citations and an H-index of 30, with an average citation of 10.37 per article.

### 3.5 Visual analysis of Co-occurring keywords

**Fig. 5:**
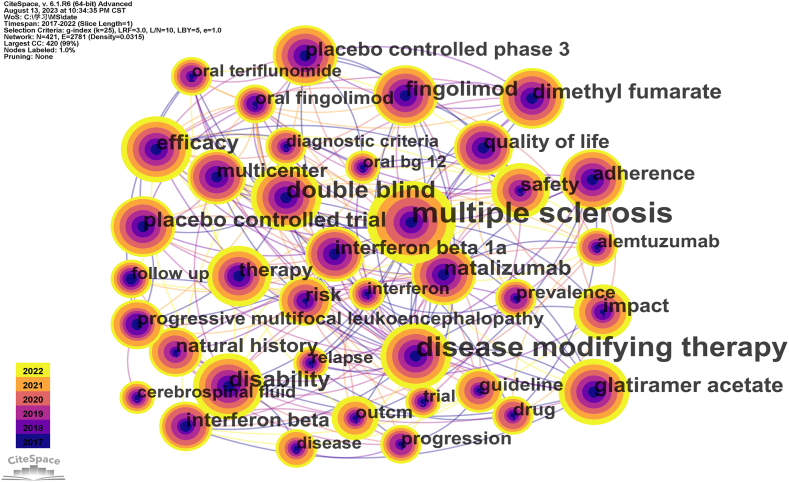
Co-occurring keywords analysis of Disease-modifying therapies for multiple sclerosis.

Keyword co-occurrence mapping reflects the research hotspots in the DMTs research field of MS (Fig. 5). As shown in Fig. 5, the nodes represent keywords, and the size of each node corresponds to the frequency of keyword co-occurrence. The top ten high frequency keywords in the past five years mainly include multiple sclerosis, disease modification therapy, double blindness, disability, natazumab, effectiveness, fingolomod, glatiramer acetate, dimethyl fumarate, and placebo-controlled study. Our analysis showed that the hottest topics in the field of research on DMTs for MS include the effectiveness of different DMTs drugs in the treatment of MS and the value of clinical applications.

### 3.6 Visual analysis of document co-citation

**Fig. 6:**
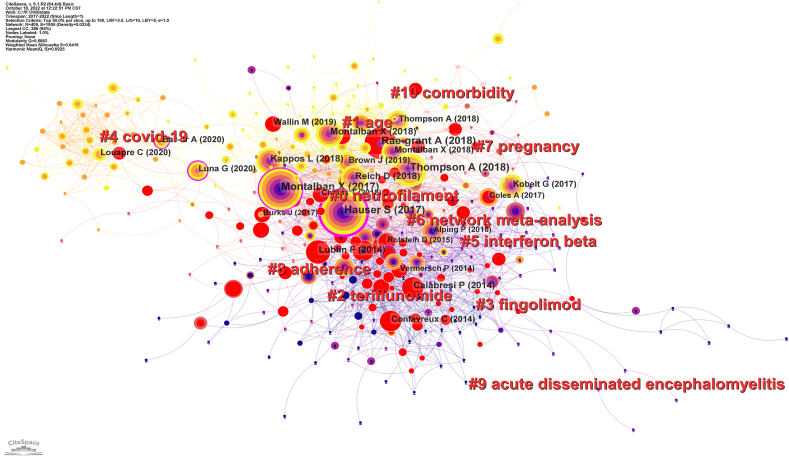
Document co-citation analysis of Disease-modifying therapies for multiple sclerosis.

Document co-citation analysis is a research method that measures the associations between different studies. It can be used to assess the research themes and development of a field in which one or more subsequent studies simultaneously cite two or more articles. Modularity *Q* and subject profile value are the two indicators used to evaluate clustering. Q > 0.3 and subject profile value > 0.5 indicates reliable and reasonable clustering results. Fig. 6 depicts the cocitation network of the literature on DMTs research in MS, containing 409 nodes, 1955 lines, and 11 major clusters. The modularity Q was 0.5883, and the average value was 0.6925. A node represents a highly cited document with the author's name marked in black. The lines represent the relationships between references and cocitations of the collected studies. Larger nodes indicated a greater number of citations. As shown in Fig. 6, the most cited literature was that published by Hauser S et al., in 2017, with 170 citations, followed by the one published by Thompson A et al., in 2018, with 161 citations, Montalban X et al., in 2017, with 130 citations, and Rae-Grant A et al., in 2018, with 126 citations. Hauser S,2017 and Montalban X, 2017, and others also have good centrality. The top ten most cited articles in the last five years are listed in Table 4.

**Table 4:** The largest 11 clusters of Disease-modifying therapy for multiple sclerosis document co-citation.

Cluster ID	Size	Silhouette	mean(Year)	Label (LLR)
0	73	0.801	2019	neurofilament
1	62	0.799	2020	age
2	53	0.804	2017	teriflunomide
3	47	0.819	2017	fingolimod
4	43	0.979	2020	covid-19
5	36	0.798	2017	Interferon beta
6	27	0.834	2018	network meta-analysis
7	23	0.920	2019	pregnancy
8	12	0.958	2018	adherence
9	5	1	2018	acute disseminated encephalomyelitis
10	4	0.995	2019	comorbidity

The article with the highest number of citations was published by Hauser S et al.; the authors randomly assigned 1656 patients with relapsing MS to either intravenous administration of ocrelizumab (600 mg every 2–4 weeks) or subcutaneous administration of interferon β-1a (44 μg three times a week) for 96 weeks to compare the therapeutic effects of ocrelizumab and interferon β-1a for MS [15]. They discovered that compared with interferon β-1a, ocrelizumab exhibited a lower rate of disease activity and progression within 96 weeks, which showed higher clinical value; however, the safety of this treatment requires further confirmation. The second most cited article is “Diagnosis of multiple sclerosis:2017 revisions of the McDonald criteria”. Thompson A et al. simplified McDonald's standard for diagnosing MS in 2010. They modified the standard by asserting that when MRI scans meet the DIS criteria, MS can be diagnosed clinically so that the McDonald standard can be better applied in clinical practice, thus increasing the early detection of MS and reducing the misdiagnosis rate [16]. In another Phase III clinical trial by MontalbanX et al., 732 patients with primary progressive multiple sclerosis were randomly assigned to the placebo and ocrelizumab groups to receive ocrelizumab (600 mg) or placebo intravenously every 24 weeks; the rate of clinical symptoms and MRI progression was lower in the ocrelizumab group than in the placebo group [17]. In addition, Montalban X has a cooperative relationship with Hauser S. and Rae-Grant A. and drafted 30 recommendations for the initiation, conversion, and termination of DMTs in patients with MS by analysing relevant evidence [18]. Kappos L et al. found that the selective S1P receptor modulator siponimod reduced the risk of disability progression in secondary progressive multiple sclerosis [19]. The definition of MS phenotypes of multiple sclerosis was published in the Journal of Neurology in 2013. This classification is still used clinically and is important for communication, prognosis, clinical trial design, recruitment, and treatment decisions [20]. Montalban et al.'s review of multiple sclerosis, published in The New England Journal of Medicine, is the highest-cited review, cited 76 times [21]. The guidelines on the pharmacological treatment of MS developed by the European Committee of Treatment and Research in Multiple Sclerosis (ECTRIMS) and the European Academy of Neurology (EAN) are the most cited, including 11 DMTs approved by the European Medicine Agency (EMA) [22]. The results of fingolimod and teriflunomide in phase 3 clinical trials for RRMS have also attracted the attention of researchers [23,24]. From the top ten highly cited articles, it can be seen that the current hotspot of DMTs in MS is still the clinical efficacy of DMT drugs, such as ocrelizumab, siponimod, fingolimod, and teriflunomide, and the clinical decision-making of DMTs treatment, as well as some diagnostic criteria and clinical classifications related to drug development and clinical decision-making.

The co-citation analysis of the literature in the field of DMTs research in MS yielded 11 clusters, labelled by the indexed keyword in the respective citations in red in Fig. 6. Cluster analysis can further extract information from the literature and help us understand current research hotspots. To characterise the nature of this clustering, the CiteSpace software extracts clustering information from article titles based on three special metrics: indexing by latent semantics, log-likelihood ratio, and mutual information, where the log-likelihood ratio provides the best results in terms of topic uniqueness and coverage associated with clustering. Table 4 summarises the details of these 11 clusters. The Silhouette profile values for each cluster were greater than 0.7, indicating reliable and meaningful results. As shown in Table 4, among the 11 clustering information, three clusters were for DMTs drugs: teriflunomide, fingolimod, and interferon β. The remaining clustering information included methodological meta-analysis and disease-specific conditions such as patient age, comorbidities, and adherence. Based on a comprehensive consideration of highly cited literature and clusters, the current research themes focus on the effectiveness of DMTs drugs in patients with MS and the strategies associated with DMT treatment.

## 4 Emerging trends

**Fig. 7:**
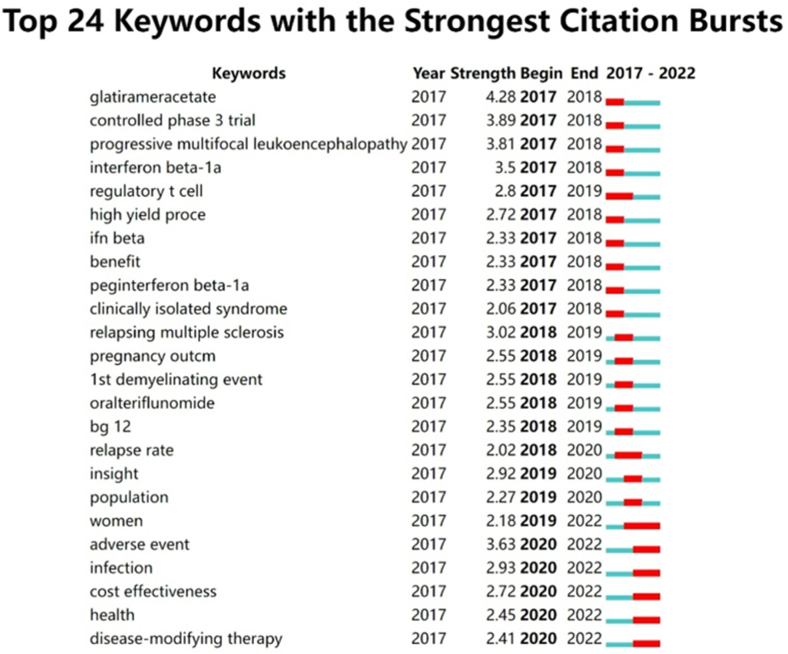
Top 24 keywords with the strongest citation burst.

Articles with citation bursts can trace the frontiers of research in the field of DMTs in MS by detecting the changing trend of keywords and references over a certain period, and not just the frequency of keywords. Fig. 7 shows the top 24 keywords with the strongest citation bursts between 2017 and 2022. Research on DMTs for MS in 2017–2018 focused on regulatory T cells, interferon, glatiramer acetate, the effectiveness of DMTs drugs, and adverse effects such as progressive multifocal leucoencephalopathy. Tiflunomide, dimethyl fumarate, and MS relapse rates have become hotspots for DMTs for MS research from 2018 to 2020; hotspots from 2020 to 2022 are mainly related to infection, adverse events, and cost-effectiveness.

**Fig. 8:**
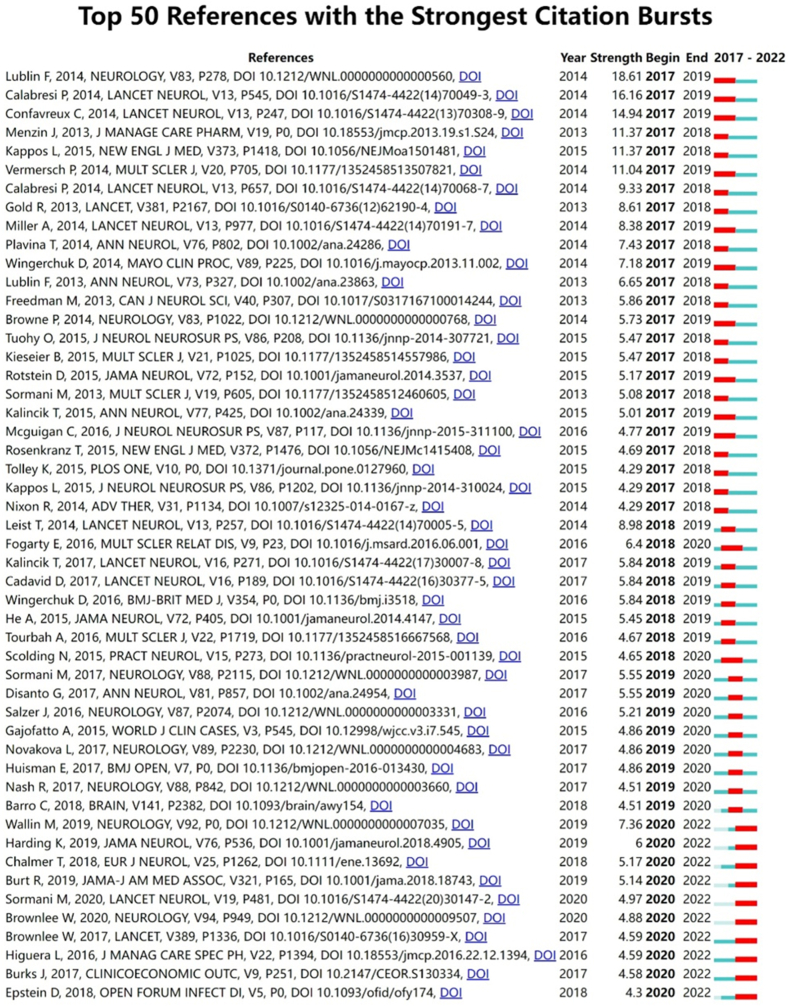
Top 50 references with the strongest citation bursts.

This is an efficient method for discovering landmark literature through citation bursts in recent literature. Fig. 8 shows the top 50 references with the highest number of citation bursts through 2022. As shown in Fig. 8, most of the citation mutations have been completed, such as glatiramer acetate and progressive multifocal leucoencephalopathy, but some are still ongoing, mainly related to topics such as complications related to the treatment of DMTs, safety, management, and treatment of MS patients. Epstein et al. summarised the risk of tuberculosis, hepatitis B virus, and herpes virus infection after DMTs in MS patients [25]. Brownle et al. explored how neurologists could manage and administer MS to patients during the COVID-19 pandemic [26]. Combined with keyword mutation analysis, the current frontiers and trends in the field of MS DMTs research still focus on the effectiveness of different DMTs drugs in treating patients with MS and methods to optimise treatment strategies. In the context of the COVID-19 pandemic, managing and rehabilitating patients with MS is also a trend for future research.

## 5 Discussion

Our study analyzed the publications of DMTs for MS and the contributions of journals, countries, institutes, and authors. Using bibliometrics to analyze highly cited literature, we drew interesting and useful conclusions, such as research hotspots, research evolutionary changes, and research trends. To the best of our knowledge, this study is the first to conduct a bibliometric and knowledge map visualization analysis of DMTs for MS research using the Web of Science database. Bibliometric analysis can help researchers grasp the current research status, development, and evolution in relevant fields, save time and energy, evaluate the research field from a macro perspective, and predict research trends.

We analyzed the number of DMT publications in the past 10 years, and the results showed that the number of articles published in MS's DMTs research field has increased rapidly in the last five years, suggesting that the field is gaining increasing attention and is now favoured by more researchers. According to the analysis of institutions and national cooperative relationship graphs, developed countries and their institutions have made substantial contributions to this field, with the United States being the largest contributor. MS affects 2–3 million people worldwide, especially minorities in the United States [21,27,28]. The annual financial burden in the United States ranges from $51,825 to $67,116 [29]. DMTs can significantly reduce the economic burden of MS in the United States [30]. The contribution of developing countries, such as China, to this field is relatively low. One possible reason is the low incidence of MS in China. The incidence of MS in China is 0.235 per 100,000 person-years, while the incidence of MS in the United States is 4.8 per 100,000 person-years [1,31]. Among the three most prolific publishing institutions, two were located in the United States (US). The top two journals, according to MS research with DMTs, were "Multiple Sclerosis, Multiple and Related Diseases." Although these journals' impact factors were lower than those of the leading journals, they held the distinction of being the largest publishers and the most cited journals. This designation positions them as authoritative sources within this field. The calculation of a journal's impact factor stems from the number of citations and articles published, but it should not be the sole criterion for gauging a journal's academic worth. The identification of highly cited journals aids readers in swiftly identifying prominent publishers frequently cited in the field, offering a reference for literature searches and manuscript submissions.

Research focal points were discerned through co-occurring keywords and document co-citations. In the domain of DMTs research on MS over the past five years, the ten most prevalent keywords encompassed "Multiple Sclerosis," "Disease Modification Therapy," "Double Blindness," "Disability," "Natalizumab," "Effectiveness," "Fingolimod," "Glatiramer Acetate," "Dimethyl Fumarate," and "Placebo-Controlled Study." Notably, the top ten highly cited articles were predominantly featured in the "New England Journal of Medicine" (3/10), "Lancet Neurology" (3/10), and "Neurology" (2/10). Singularly, one article was published within a journal dedicated to multiple sclerosis. Half of these articles constituted randomized controlled trials (RCTs) probing the efficacy of DMTs, including ocrelizumab, fingolimod, teriflunomide, and siponimod. The efficacy and safety of DMTs are pivotal for subsequent research and clinical endeavors. Furthermore, literature featured in premier journals is more likely to garner attention and recognition from researchers.

Noteworthy is the absence of familiar DMT drugs such as natalizumab, glatiramer acetate, and dimethyl fumarate in the realm of highly cited literature. Examination of keywords displaying robust citation bursts unveiled that bursts for glatiramer acetate and dimethyl fumarate terminated in 2018 and 2019, respectively. A scrutiny of keywords exhibiting substantial citation bursts revealed a burgeoning interest among researchers in progressive multifocal encephalopathy triggered by DMT drugs. Notably, an augmented risk of progressive multifocal leucoencephalopathy (PML) in MS patients appeared linked to natalizumab and dimethyl fumarate [32,33]. The heightened risk of progressive multifocal leucoencephalopathy (PML) linked to natalizumab underscores the need for comprehensive management strategies, including diligent monitoring of the anti-JC virus (JCV) antibody index and PML stratification, coupled with MRI screening [34]. In 2018, citation bursts for daclizumab came to an abrupt halt, coinciding with its discontinuation [35].The withdrawal of daclizumab in 2018 resulted from the emergence of additional, albeit rare, serious autoimmune adverse effects, notably immunomediated encephalitis [36].

Moreover, a scrutiny of keywords and references with the most robust citation bursts unveiled a trending focus on the interplay between MS and COVID-19 infection, as well as strategies for mitigating the epidemic's adverse impact on MS patients. Individuals with MS undergoing DMTs face an augmented susceptibility to infections, encompassing S1P1 modulators and ocrelizumab [26,37]. A fresh area of interest for researchers revolves around the safety and efficacy of SARS-CoV-2 vaccines in patients with MS receiving DMTs. It is worth noting that select DMTs may diminish the serological response to SARS-CoV-2 vaccination in MS patients [38,37]. For instance, S1P1 modulators and CD20 therapies have demonstrated a reduction in the real-world effectiveness of COVID-19 vaccinations [39]. The risk of infection related to MS treatment has become a pivotal consideration in therapy selection [40]. Consequently, future research endeavors should elucidate the impact of COVID-19 on MS treatment and delve into the development of DMTs with a lower risk of infection-related adverse effects.

This study has some limitations. First, it is a citation-based bibliometric analysis, and most of the results depend on data extracted from the literature. The extracted data included the authors, institutions, countries, keywords, and citations. For the content of the literature, the main body of knowledge we want to acquire, bibliometrics, cannot be extracted or analyzed. It could not replace literature reading; therefore, the information collected was limited, and the results may not be persuasive. Second, highly cited literature cannot fully represent high-quality literature, nor can it fully represent high-impact literature. A comprehensive evaluation of the impact of the literature should be conducted. In addition to the number of citations, the academic value of an article should also be considered, including whether it has added new content to existing knowledge and whether it can guide the clinical management of diseases. The impact factor, H-index, reputation, and self-citation rate of a journal should also be considered. Furthermore, the total number of citations may be significantly affected by the age of the literature, which may lead to bias in screening highly cited articles. Third, the limited number of keywords, burst detection, and time plots of keywords may not represent the true hotspots. Finally, this study only analyzed relevant research in the Web of Science database, owing to the inherent limitations of the CiteSpace software, and the research results have certain deviations. Despite these drawbacks, the visual analysis of citations is the most widely used and intuitive method in bibliometric analysis [41].

## 6 Conclusion

This study utilized quantitative scientometric methods to investigate the trends in knowledge and treatment related to DMTs in MS research. By reviewing previous studies in the field, the study predicted future research perspectives. The findings indicate a significant and sustained increase in DMTs for MS research over the last decade. The analysis of highly cited literature and keywords unveiled the evolution of research topics and patterns in DMTs for MS, highlighting areas currently experiencing rapid growth. The study also expanded its scope to include recent topics such as the adverse effects of medication and the treatment of COVID-19 in MS patients. Notably, the study suggests promising performance of DMT strategies in MS patients based on scientometric analysis results, indicating potential future attention. The insights gained from this study can aid professionals in comprehending evolutionary modes and trends in the field.

## Funding

This work has been co-financed by Research project of Traditional Chinese Medicine in Gansu province [GZKZ-2021-9]; Cuiying Scientific and Technological Innovation Program of' The Second Hospital & Clinical Medical School, 10.13039/100012899Lanzhou University (CY2022-MS-A17 and CY2023-QN-B02); The Science and Technology Plan Project of Chengguan District of Lanzhou City (2019RCCX0068) and Lanzhou Talent Innovation and Entrepreneurship Project Foundation of China (2021-RC-98). The funders had no role in study design; collection, analysis, and interpretation of data; in the writing of the report; and in the decision to submit this article for publication.

## Data availability statement

Data will be made available on request.

## CRediT authorship contribution statement

**Ting Zheng:** Data curation, Formal analysis, Funding acquisition, Investigation, Methodology, Project administration, Resources, Software, Supervision, Validation, Visualization, Writing – original draft, Writing – review & editing. **Taotao Jiang:** Data curation, Formal analysis, Investigation, Software, Validation, Visualization, Writing – original draft. **Zilong Huang:** Data curation, Formal analysis, Software, Visualization. **Manxia Wang:** Conceptualization.

## Declaration of competing interest

The authors declare that they have no known competing financial interests or personal relationships that could have appeared to influence the work reported in this paper.
